# Quantitative Image-Based Cell Viability (QuantICV) Assay for Microfluidic 3D Tissue Culture Applications

**DOI:** 10.3390/mi11070669

**Published:** 2020-07-09

**Authors:** Louis Jun Ye Ong, Liang Zhu, Gabriel Jenn Sern Tan, Yi-Chin Toh

**Affiliations:** 1 Department of Biomedical Engineering, National University of Singapore, 4, Engineering Drive 3, E4-04-10, Singapore 117583, Singapore; louis.ongjunye@qut.edu.au (L.J.Y.O.); zhu_liang@simtech.a-star.edu.sg (L.Z.); gabriel.tanjs@gmail.com (G.J.S.T.); 2 Institute for Health Innovation and Technology, National University of Singapore, 14 Medical Drive, #14-01, Singapore 117599, Singapore; 3 School of Mechanical, Medical and Process Engineering, Queensland University of Technology (QUT), Level 7, O Block, Gardens Point Campus, Brisbane City QLD 4000, Australia; 4 Singapore Institute of Manufacturing Technology, 31 Biopolis Way, #04-10 Nanos, Singapore 138669, Singapore; 5 The N.1 Institute for Health, 28 Medical Drive, #05-corridor, Singapore 117456, Singapore; 6 NUS Tissue Engineering Programme, National University of Singapore, 28 Medical Drive, Singapore 117456, Singapore; 7 Institute of Health and Biomedical Innovation, Queensland University of Technology (QUT), Q Block-IHBI, 60 Musk Avenue, Kelvin Grove QLD 4059, Australia

**Keywords:** 3D cell culture, microfluidic, cell viability, quantitative, tissue culture

## Abstract

Microfluidic 3D tissue culture systems are attractive for in vitro drug testing applications due to the ability of these platforms to generate 3D tissue models and perform drug testing at a very small scale. However, the minute cell number and liquid volume impose significant technical challenges to perform quantitative cell viability measurements using conventional colorimetric or fluorometric assays, such as MTS or Alamar Blue. Similarly, live-dead staining approaches often utilize metabolic dyes that typically label the cytoplasm of live cells, which makes it difficult to segment and count individual cells in compact 3D tissue cultures. In this paper, we present a quantitative image-based cell viability (QuantICV) assay technique that circumvents current challenges of performing the quantitative cell viability assay in microfluidic 3D tissue cultures. A pair of cell-impermeant nuclear dyes (EthD-1 and DAPI) were used to sequentially label the nuclei of necrotic and total cell populations, respectively. Confocal microscopy and image processing algorithms were employed to visualize and quantify the cell nuclei in the 3D tissue volume. The QuantICV assay was validated and showed good concordance with the conventional bulk MTS assay in static 2D and 3D tumor cell cultures. Finally, the QuantICV assay was employed as an on-chip readout to determine the differential dose responses of parental and metastatic 3D oral squamous cell carcinoma (OSCC) to Gefitinib in a microfluidic 3D culture device. This proposed technique can be useful in microfluidic cell cultures as well as in a situation where conventional cell viability assays are not available.

## 1. Introduction

Microfluidic cell culture platforms have gathered growing attention to perform in vitro drug testing studies due to their ability to control cell microenvironments while utilizing a small amount of samples [1]. Typically, a microfluidic cell culture device consists of fluidic networks ranging from 10 to 500 µm, which serve as connections to chambers housing different cell types [2,3,4,5,6,7], with fluid volumes ranging from nanolitres to microlitres [8,9]. The small geometries also allow for the establishment of 3D tissue constructs using a small number of cells (100–10,000 cells) [10]. This miniaturization capability facilitates an increase in throughput for drug testing applications without an appreciable increase in the sample cost. However, the practical translation of microfluidic cell culture devices for drug testing applications must be complemented with cellular assays, which can be implemented in a format compatible with microfluidic devices. The cell viability measurement is a common biological readout used to evaluate drug responses in microfluidic systems, although the minute cell number and liquid volume impose significant technical challenges when performing quantitative cell viability measurements using conventional methods [11]. For instance, bulk cell viability assays, such as MTS and Alamar Blue, rely on the conversion of a substrate into colored or fluorescent products by cytoplasmic metabolic enzymes in living cells, which are then collected in the culture medium and measured using spectrophotometry. Medium perfusion coupled with the minute cell number in microfluidic devices often result in low concentrations of metabolic products, which fall below the detection limit of many existing spectrophotometers [11]. Therefore, most commercial cell viability assay kits cannot detect fluorescence/colorimetric signals with microfluidic cell culture devices.

An alternative method of determining cell viability in microfluidic devices is an imaging approach, whereby live and dead cell populations are labeled with a pair of metabolic and nucleic acid dyes, often known as live-dead staining [12,13]. The metabolic probe, such as Calcein AM and Resazurin, will distinguish live cells from the ability of cytoplasmic metabolic enzymes to generate fluorescent products; while the cell-impermeant nucleic acid dye, such as Propidium Iodide, will selectively label the nuclei of necrotic cells whereby the cell membrane is no longer intact. As more microfluidic devices are designed to support the 3D tissue culture by incorporating either microstructures that physically pack cells at high density [12,14,15]; cell-laden hydrogels [16]; scaffolds [17]; or micropatterning [18] to improve physiological relevance [2,4,5,12,19,20], it becomes increasingly challenging to segment and quantify individual cells within a compact 3D cell mass during image processing to get quantitative readouts [14,21]. Thus, this approach is mainly limited to the qualitative assessment of cell viability in microfluidic cell cultures [12], which does not provide the level of sensitivity required to discern between subtle changes in cell viabilities across different drug concentrations or between cell types with differential drug responses.

Here, we propose the quantitative image-based cell viability (QuantICV) assay to provide a quick and reliable method for measuring cell viability in microfluidic 3D cell cultures. An imaging-based readout allows us to track the cell viability status at a single-cell resolution, and therefore, overcome the limitation of having the minimum cell number required in bulk cell viability assays. Instead of employing metabolic live cell dyes, which label the entire cell cytoplasm, a pair of cell-impermeant nucleic acid dyes was administered in sequential order to label the necrotic and total cell populations. By selectively labeling the cell nuclei, which occupy ~10% of the cell volume [22], individual cells can be spatially resolved even in tightly-packed 3D cell aggregates. When combined with confocal microscopy and image processing, we were able to accurately quantify the number of living and dead cells within 3D spheroids or aggregates at a single cell resolution. This imaging-based cell viability quantification method has been validated against a conventional MTS assay with good concordance in both 2D and 3D cultures. Finally, we employed the QuantICV assay to determine the differential drug response of parental and metastatic 3D microfluidic oral squamous cell carcinoma (OSCC) models.

## 2. Materials and Methods 

### 2.1. Materials

All materials and reagents were purchased from Sigma-Aldrich Pte. Ltd., Singapore, unless otherwise stated.

### 2.2. Device Design, Fabrication, and Sterilization

The microfluidic device was modified from a previous study [5]. Briefly, the microfluidic channel dimensions were 1 cm (length), 600 µm (width), and 100 µm (height). The channel was divided into a 200-µm wide central cell culture compartment, which is flanked by two perfusion compartments, by a micropillar array. The micropillar array was made up of 30 × 50 µm parallelogram pillar structures separated by 20 µm gaps. Each perfusion compartment was 200 µm wide.

The microfluidic devices were fabricated by molding polydimenthylsiloxane (PDMS) (Dow Corning, USA) on DRIE-etched silicon templates (Data Storage Institute, Singapore) [4] (Supplementary Figure S2). The microfluidic devices were capped with PDMS substrate-coated glass coverslips with a plasma cleaner (FemtoScience, Nashville, TN, USA) at 50 W, 20 sccm of O_2_ for 50 s. A process flow chart for device fabrication is provided in Supplementary Figure S3.

The perfusion inlet and outlet of the devices were connected to 3-mL fluid dispensing reservoirs (Nordson EFD, Westlake, OH) using Optimum^®^ angled dispense tips (Nordson EFD, Westlake, OH, USA) and one-way stopcocks with Luer connections (Cole-Parmer, Vernon Hills, IL, USA). All the parts were sterilized by autoclaving at 121 °C for 20 min except for the stopcocks, which were disinfected by soaking with 70% ethanol for 1 h. Sterilized devices were first primed by subjecting the devices in a 1 X PBS bath in vacuum for 30 min. Surface passivation of the primed devices were conducted by pipetting 2% Pluronic^®^ F-127 into the device for 1 h to prevent cell adhesion. The devices were assembled and mounted on 3D printed support frames printed using an Objet260 Connex3 Printer (Stratasys, Eden Prairie, MN, USA) with an acrylonitrile butadiene styrene (ABS) plastic.

### 2.3. Maintenance of Patient-Derived (HN137) OSCC and Spheroid Formation

Patient-derived (HN137) primary and metastatic oral squamous cell carcinoma (OSCC) cell lines were gifts from Dr. Ramanuj DasGupta (Genome Institute of Singapore, Singapore). Both the HN137 cell lines were established from a patient with synchronous primary and metastatic tumors, as previously reported [23]. The cell lines were routinely maintained in a RPMI 1640 medium (Life Technologies, Singapore) supplemented with a 10% fetal bovine serum (FBS) and penicillin-streptomycin (10,000 U/mL). To form tumor spheroids, confluent cells were harvested using 1.25% Trypsin-EDTA (Life Technologies, Singapore) and seeded into Aggrewell400™ plates (StemCell Technologies, Vancouver, Canada) at a density of 83 cells/microwell and incubated for 24 h. The culture volume was 2 mL per culture well.

### 2.4. Seeding and Perfusion Culture of Patient-Derived (HN137) OSCC in Microfluidic Devices

HN137 spheroids were seeded into the device by withdrawing the spheroid suspension from the cell seeding inlet through the common outlet with a syringe pump (KD Scientific, Holliston, MA, USA) at a flow rate of 0.03–0.08 mL/h. Once the cell culture compartment was filled with cells, the cell seeding inlet and the common outlet were closed off. The outlet reservoir was connected to the stopcock at the common outlet and medium perfusion was initiated by turning on the perfusion inlet and the common outlet of the device. The seeded device was placed inside a sterile polypropylene container and maintained in a 5% CO_2_ incubator. The perfusion inlet was topped up every 24 h by manually transferring the medium from the outlet.

### 2.5. Dose-Response Study of Patient-Derived (HN137) OSCC Cultures to Gefitinib

A 2D dose-dependent study of both parent and metastatic HN137 cancer cells were conducted by seeding 30,000 cells into 96-well plates (ThermoFisher Scientific, Waltham, MA, USA) and allowed to attach for 1 day. Attached cells were exposed to 200 µL of a Gefitinib working solution at a concentration of 0, 0.05, 0.1, 1.33, 8, 13.33, 16, 18.67, and 20 µM for four days. Medium change was performed at a two-day interval.

A 3D spheroid dose-dependent study of both the parental and metastatic HN137 cancer cells were conducted using Aggrewell400™ plates (StemCell Technologies, Vancouver, VAN, Canada). The seeded cells were cultured in the Aggrewell400™ plates for one day to allow the formation of spheroids. Following that, the spheroids were exposed to 2 mL of a Gefitinib working solution at a concentration of 0, 0.05, 0.1, 1.33, 8, 13.33, 16, 18.67, and 20 µM for four days. Medium change was done every two days.

The dose-dependent study of 3D parental and metastatic HN137 cancer cells in the microfluidic device was conducted by first culturing the seeded spheroids within the microfluidic device for one day followed by changing out the medium reservoir to a Gefitinib-containing medium at a concentration of 0, 0.05, 0.1, 1.33, and 16 µM for four days. A manual top-up of the culture medium was conducted daily by pipetting the flow-through collected at the outlet reservoir to the inlet reservoir.

### 2.6. Quantitative Image-Based Cell Viability (QuantICV)

A 2D image-based cell viability quantification staining was conducted by incubating the cell cultures in 4 µM of EthD-1 (ethidium homodimer-1) (Life Technologies, Singapore) in a cell culture medium for 20 min followed by washing with 1 X PBS and sample fixation with 4% paraformaldehyde (PFA) for 20 min. The fixed cultures were permeabilized with 0.5% Triton X-100 in a PBS solution, and blocked with a blocking buffer (2% BSA/PBS) before counter-staining with 1 µg/mL of DAPI (4′,6-diamidino-2-phenylindole) for 20 min. The stained wells were washed with 1 X PBS before imaging.

A 3D image-based cell viability quantification staining was conducted by first incubating the spheroids with collagenase at 0.05 µg/mL in PBS for 20 min. After washing with a culture medium, the spheroids were stained with 4 µM of EthD-1 in a cell culture media for 2 h followed by washing with 1 X PBS and sample fixation with 4% paraformaldehyde (PFA) for 20 min. The fixed cultures were permeabilized with 0.5% Triton X-100 in a PBS solution for 30 min and blocked with a blocking buffer (2% BSA/PBS) before staining with 1 µg/mL of DAPI for 2 h. The stained spheroids were washed with 1 X PBS before imaging.

To stain the 3D microfluidic cultures, the outlet of the microfluidic device was connected to a withdrawal syringe pump. The inlet fluids were replaced with collagenase at 0.05 µg/mL in PBS and withdrawn into the microfluidic device at 0.1 mL/h for 20 min. This was followed by a culture medium for washing for 20 min at the same flow rate. The collagenase-treated 3D cultures were subsequently perfused with 4 µM of EthD-1 in a cell culture media for 2 h followed by 30 min of 1 X PBS, 1 h of 4% paraformaldehyde (PFA) for cell fixing, and 2 h of 1 µg/mL of DAPI. The stained 3D cultures were perfused with 1 X PBS to wash off any staining residues for 15 min before imaging.

All samples were imaged using a confocal microscope (LSM 800, Zeiss, Germany). For 3D spheroid cultures, the spheroids were harvested and placed onto a custom-made 200 µm thick PDMS gasket before placing on the microscope stage. Full-volume imaging of the 3D spheroids was conducted at a slice interval of 5 µm. Representative slices of the 3D spheroids were obtained after the full-volume imaging by imaging at half the spheroid depth. For 3D microfluidic cultures, optical sections spanning the middle of the 3D tissues were obtained. This was achieved by manually determining the tissue thickness using the live mode and inputting the depth equivalent to half of the tissue thickness. Image analysis and visualization were performed using the Imaris 9.0 (Bitplane, Zurich, Switzerland) in-built spot function with a spot size threshold set to 6 µm. The viable cell population was calculated based on the ratio of necrotic cells (stained positive for ETHD-1) to total cell count (stained positive for DAPI) as follows:

$$\left(1-\frac{Number\;of\;EthD^{+}cells}{Number\;of\;DAPI^{+}cells}\right)\times 100\%$$

### 2.7. Statistical Analysis

The results were obtained from at least three independent experiments. The values were expressed as the mean ± standard deviation (SD). Data fitting were done with GraphPad Prism 8 (GraphPad, San Diego, CA, USA) using a four-parameter logistic regression. A two-way ANOVA was used for statistical significance of the data between the parental cultures and metastatic cultures using GraphPad Prism 8. Values with *p* < 0.05 were considered statistically significant.

## 3. Results

### 3.1. Working Principle of the Quantitative Image-Based Cell Viability (QuantICV) Assay

**Figure 1:**
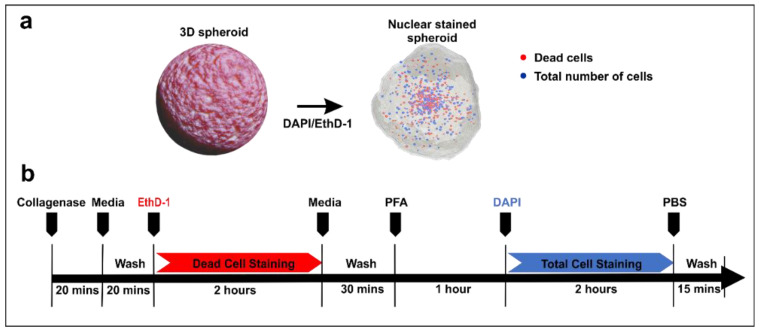
Working principle and procedures in the quantitative image-based cell viability (QuantICV) assay. (**a**) A pair of cell-impermeant nucleic acid dyes (EthD-1/DAPI) was used to selectively label the nuclei of necrotic and total cell population. (**b**) Cell labeling procedure. The cells were first stained with EthD-1 to mark the existing dead cell population followed by sample fixation and staining with DAPI to label the entire cell population.

Metabolism-based viability dyes often label the entire cell cytoplasm, which makes it difficult to segment individual cells in a 3D spheroid [21,24]. To circumvent this limitation, we propose to rely on nuclear staining to identify individual cells within densely-packed 3D cell cultures. It has been estimated that the mammalian cell nucleus occupies approximately 10% of the entire cell volume [22]. Here, we chose to employ cell-impermeant nuclear dyes, which cannot pass through the cell membrane and therefore only selectively label dead or fixed cells. A pair of cell-impermeant nuclear dyes was applied to the 3D cellular construct in sequential order (Figure 1). This strategy decouples optimization of staining conditions for the two dyes to ensure a good signal-to-noise ratio during image acquisition. For this study, we used the EthD-1 (Ethidium homodimer-1)/DAPI (4′,6-diamidino-2-phenylindole) nuclear dye pair as a proof of concept. The EthD-1 dye is commonly used to identify dead cells due to their inability to permeate intact membrane [13], while DAPI is commonly used to identify the presence and location of cells in cell cultures [12]. The first nuclear dye (EthD-1) would label the existing dead cell population, while the second dye (DAPI) would label the total cell population after the entire 3D cell construct was fixed (Figure 1a). Since DAPI and EthhD-1 have different preferred binding sites to dsDNA [25,26], this reduces the competitive binding of the selected dyes within the cell nucleus, and hence do not compromise the fluorescence signals emanating from both nuclear dyes. Consequently, the EthD-1/DAPI nuclear dye pair staining resulted in consistent fluorescence signals for quantifying the dead cell number and the total number of cells in the culture. In order to ensure more uniform dye penetration across the 3D cell construct, we treated all 3D cell constructs with collagenase [27] prior to the nuclear staining steps (Figure 1b). 

### 3.2. Validation of the QuantICV Assay in a Conventional 2D Cell Culture

To assess the accuracy of the QuantICV assay in measuring cell viability, we validated it against a conventional bulk MTS assay in 2D cultures to determine the dose responses of patient-derived parental and metastatic oral squamous cell carcinoma (OSCC) tumor cell lines to Gefitinib. OSCC is one of the most common types of oral cancer [28], and can manifest in various sub-types that have differential responses to anti-cancer drugs, including Gefitinib [23,29]. Gefitinib is an EGFR inhibitor used to treat recurrent head and neck cancers. In this study, we used a pair of OSCC cell lines, which were derived from a single patient with synchronous primary and metastatic tumors, to evaluate the sensitivity of the assay in measuring differential drug response between different OSCC sub-types.

**Figure 2:**
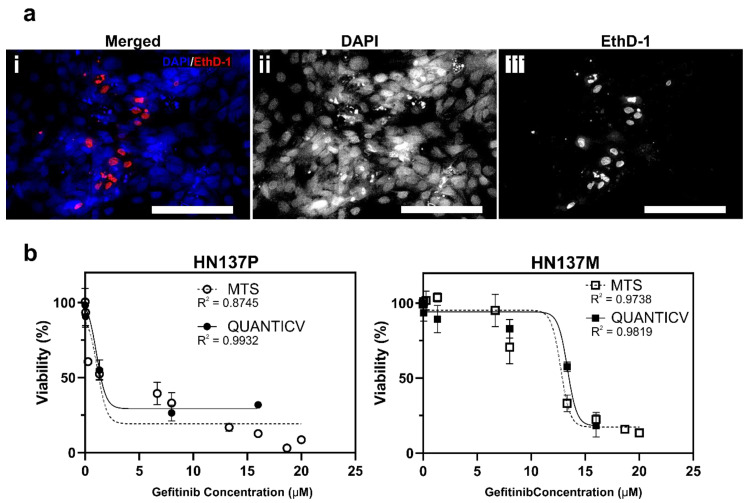
Validation of the QuantICV assay against the MTS assay in two-dimensional (2D) cultures of parental (HN137P) and metastatic (HN137M) OSCCs. (**a**) Confocal fluorescent images showing nuclei staining of dead (EthD-1) and total (DAPI) cell populations in 2D oral squamous cell carcinoma (OSCC) cultures. Images presented were selected from a stitched, tiled image across multiple sections of the culture well presented in Supplementary Figure S3a. Scale bars = 100 µm. (**b**) Quantification of cell viabilities in parental and metastatic HN137 cells after treatment with various Gefitinib concentrations using the MTS assay (solid line) and QuantICV assay (dotted line). Data are averages of *n* = 3 repeats with ± deviation (SD).

For 2D cultures, the nuclei of individual cells labeled by the two nuclear stains can be easily identified and quantified by image processing in confluent cultures (Figure 2a). The accuracy of the QuantICV assay in predicting drug dose-responses can be evaluated by comparing the half maximal inhibitory concentration (IC_50_) values to that obtained using the MTS assay (Figure 2b). The QuantICV assay measured similar dose-response behaviors of the HN137 OSCC lines across a wide range of drug concentrations. The predicted IC_50_ of Gefitinib on parental OSCC using QuantICV was 1.06 µM, whereas the MTS assay predicted 1.14 µM. For the metastatic OSCC, QuantICV predicted an IC_50_ of 13.36 µM whereas the MTS assay predicted 12.74 µM. Hence, there was concordance in the IC_50_ values obtained using both the QuantICV and MTS assays. Using a four-parameter data fitting, similar trends in the drug response were observed, with high R-squared values indicating a good fit. From this observation, we conclude that the QuantICV assay can be an alternative technique for cell viability measurement.

### 3.3. Validation of the QuantICV Assay in Dense-Packed 3D Cell Cultures

**Figure 3:**
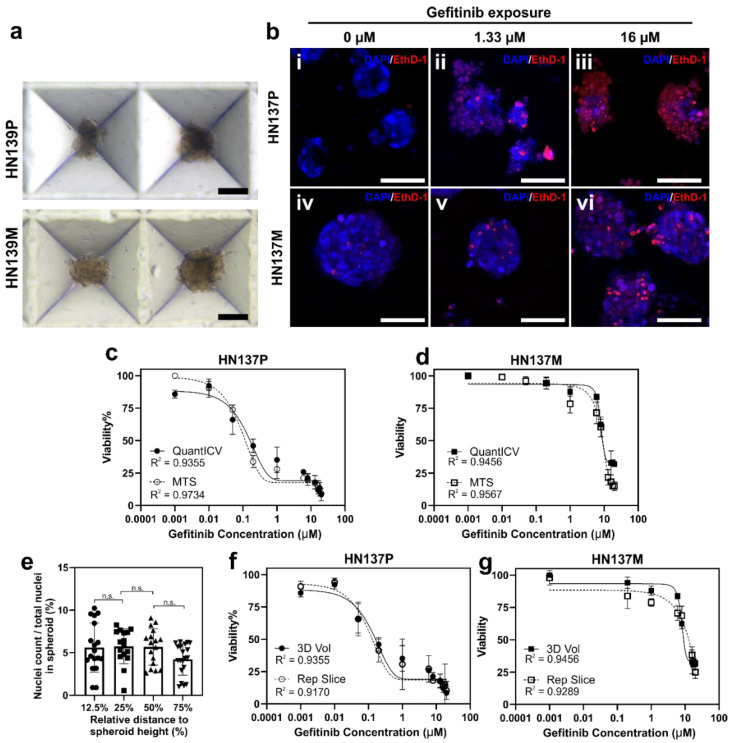
Validation of the QuantICV assay against the MTS assay in three-dimensional (3D) spheroid cultures of parental (HN137P) and metastatic (HN137M) OSCCs. (**a**) Brightfield images of parental OSCC (HN137P) and metastatic OSCC (HN137M) spheroids. Scalebar = 100 µm. (**b**) Confocal images showing double nuclear staining in (i–iii) parental (HN137P) and (iv–vi) metastatic (HN137M) OSCC spheroids exposed to different concentrations of Gefitinib. Scalebar = 100 µm. (**c**,**d**) Comparison of dose-responses to Gefitinib measured by MTS and QuantICV assays in (**c**) parental (HN137P) and (**d**) metastatic (HN137M) OSCCs. Data are averages of *n* = 3 repeats with ± standard deviation (SD). (**e**) Box plot showing cell nuclei distribution in 3D tumor spheroids at 12.5%, 25%, 50%, and 75% of spheroid depth. Uniform distribution of cell nuclei observed within 12.5–75% of the cell volume. *p* > 0.05. (**f**,**g**) Comparison of dose-responses to Gefitinib measured by enumerating dead and total cell nuclei in the entire 3D spheroid volume (3D Vol) or a single cross-sectional image slice (Rep Slice) in (**f**) parental (HN137P) and (**g**) metastatic (HN137M) OSCCs. Data are averages of *n* = 3 repeats with ± standard deviation (SD).

The QuantICV assay was developed to allow quantification of the cell number in dense-packed 3D cell cultures. Hence, we evaluated the assay in 3D spheroid cultures. Both the parental and metastatic OSCCs were established as 3D spheroids (Figure 3a) using previously established methods [5]. We noted that the staining protocol could effectively label the cell nuclei in the entire volume of the 3D spheroid (Figure 3b). This staining uniformity indicated that the dye staining protocol is suitable for densely packed tissue cultures. There was a good agreement in drug responses measured by the QuantICV and MTS assays based on the dose-response curves and calculated IC_50_ values (Figure 3c,d). Using the MTS assay, we determined that the IC_50_ of parental OSCC to Gefitinib was 0.23 µM, while the value determined from QuantICV was 0.18 µM (Figure 3c). For metastatic OSCC, the MTS assay reported an IC_50_ of 8.2 µM, while QuantICV reported 8 µM (Figure 3d). Similar to the MTS assay, the QuantICV was able to detect the increased sensitivity of both parental and metastatic OSCCs to Gefitinib in 3D cultures as compared to 2D cultures (Figure 2b,c), which was reflected by the decrease in IC_50_ values from ~1 to 0.2 µM (parental) and ~13 to 8 µM (metastatic). The results suggested that the QuantICV assay showed similar sensitivity as the MTS assay with as little as 83 cells per spheroids.

Given that the nuclear dyes could uniformly label cells in the entire 3D spheroid volume (Figure 3b), we attempted to simplify the image acquisition and processing procedures to reduce the assay time and increase throughput. A full volume 3D reconstruction of a 100 µm diameter spheroid (with 5 um z-sections) using confocal microscopy would require 20 repeated scans, which typically took around 60 min. We wanted to determine whether cell viability quantified from a single image slice can be representative of the entire spheroid. First, we confirmed that we can uniformly label and count cell nuclei at different depths of the 3D spheroid (Figure 3e). Next, cell viability was quantified from a single cross-sectional image obtained at 50% of the spheroids’ height and compared to that obtained from reconstructed 3D spheroid volumes. We observed that this simplified representative cross-section approach was able to obtain drug dose-response curves and IC_50_ values, which were similar to the full-volume approach (Figure 3f,g). For subsequent experiments, this modified QuantICV assay was adopted to accommodate drug screening with a higher throughput.

### 3.4. Dose-Response Study Using the QuantICV Assay in Microfluidic 3D Cell Cultures

**Figure 4:**
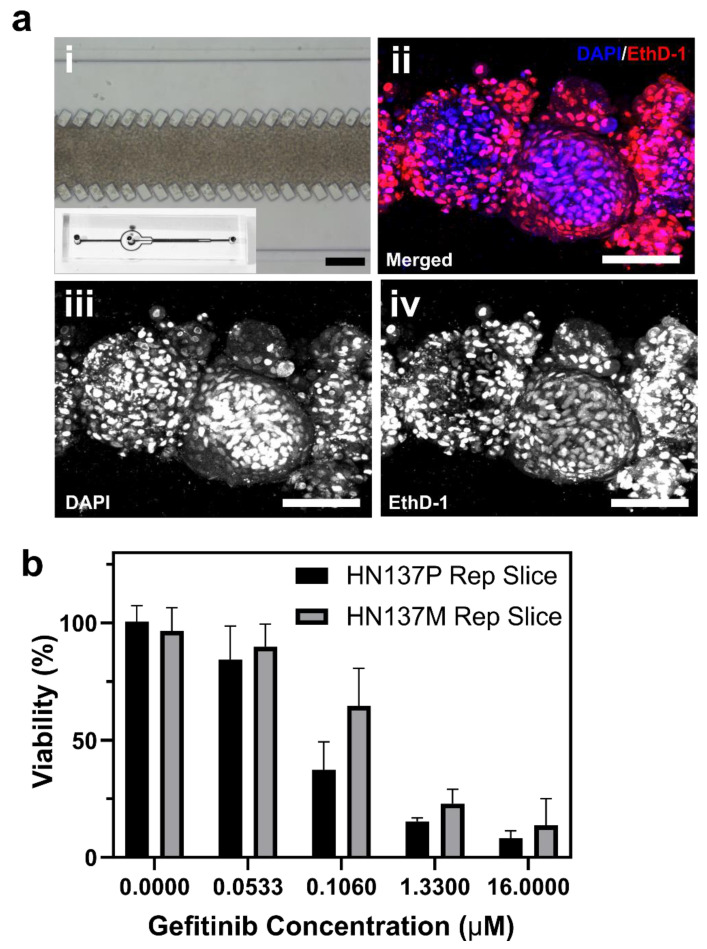
Evaluation of the QuantICV assay in quantifying cell viability in microfluidic 3D cell cultures. (**a**) Images showing 3D cultures of patient-derived (OSCCs) in microfluidic devices. (**i**) Transmission image showing a densely packed 3D tumor aggregate in the central compartment of the microfluidic device (inset). (**ii**–**iv**) Confocal images of patient-derived parental (HN137P) after double nuclear labeling, (**ii**) merged, (**iii**) DAPI^+^, (**iv**) EthD-1^+^. Cells were exposed to 1.33 µM of Gefitinib cultured within microfluidic perfusion devices. Scale bars = 100 µm. (**b**) Cell viabilities of parental (HN137P) and metastatic (HN137M) OSCCs after Gefitinib treatment using the QuantICV assay. Data are averages of *n* ≥ 3 devices ± standard deviation (SD).

Here, we evaluated the utility of the QuantICV assay in quantifying cell viability in microfluidic 3D cell cultures. We incorporated the parental and metastatic HN137 OSCC spheroids into a previously reported microfluidic device, which contained a micropillar array for packing single cells or cell aggregates at high density [5] (Figure 4a,b). Different concentrations of Gefitinib were perfused through the device for four days before the modified ICV assay was used to determine cell viability. Both the parental and metastatic HN137 OSCCs were able to remodel into a continuous packed 3D tumor aggregate in the microfluidic device after 24 h of perfusion culture (Supplementary Figure S1). After sequential perfusion with the dual nucleic acid dyes, single cross-sectional images were acquired at half of the tissue height (~50 µm) (Figure 4a ii–iv). It was observed that there was uniform staining across the 3D tumor aggregate, demonstrating that the QuantICV assay can be implemented in perfusion microfluidic devices with similar results as compared to the static 3D cultures.

At very low (i.e., 0.05 µM) or very high (i.e., 16 µM) drug concentrations, parental and metastatic OSCC cultured in the microfluidic device were equally non-responsive or susceptible to the Gefitinib treatment. Cell viabilities of parental and metastatic OSCC were 84 ± 9.1% and 90.1 ± 5.3% respectively, at 0.05 µM of the drug treatment (Figure 4c,d). At 16 µM, parental and metastatic OSCC had a cell viability of 8.23 ± 2.6% and 13.69 ± 9.3%, respectively (Figure 4c,d). These results were consistent with those in the static 3D spheroid cultures (Figure 3c,d). At intermediate drug concentrations (0.1 and 1.33 µM), the two tumor cell lines exhibited a predicted differential drug response, whereby metastatic OSCC had a significantly higher cell viability than parental OSCC (Figure 4b). However, we also observed a significant lower estimated cell viability in the microfluidic cultures when compared to the static 3D spheroid cultures (Figure 4b). For example, at 1.33 µM Gefitinib, cell viabilities in microfluidic devices were at 15.3 ± 1.4% and 22.9 ± 6% for parental and metastatic OSCC while 3D spheroid models measured 35.1 ± 5.7% and 88 ± 9.5%, respectively. The results indicated that both the tumor cell lines exhibited increased sensitivity to the drug treatment when maintained in a microfluidic perfusion device. This is supported by previous studies showing that tumor cells under a continuous perfusion culture exhibited increased sensitization towards the drug treatment via the enhanced mass transport and mimicking tissue microenvironment [30,31]. We also noted that without a collagenase treatment prior to the staining process, it is difficult to achieve consistently uniform dye penetration within the microfluidic devices (Supplementary Figure S4). Using a two-way ANOVA, we confirmed that there were differences in drug response between the parental and metastatic cell lines (*p* = 0.0175).

## 4. Discussion

The QuantICV assay was designed specifically to enable quantification of cell viability in a small amount of 3D tissue samples, which are typically found in microfluidic devices developed for organs-on-chip applications [7,32,33,34,35,36,37]. An imaging-based approach circumvents limitation in the detection of colorimetric or fluorogenic metabolites generated by cells in traditional bulk cell viability assays. As compared to existing live-dead staining methods, e.g., Calcein AM-EthD-1 [11] or Fluorescein diacetate (FDA)-propidium iodide (PI) [26], which often use a fluorescent metabolic dye to label live cells and a nucleic acid dye to label dead cells, the QuantICV assay utilizes a pair of cell-impermeant nucleic acid dyes to denote the viability status of cells. Nucleic acid dyes are low molecular weight dyes, which can permeate through cells much more quickly than metabolic dyes. This enables one to uniformly label the entire cell population in a densely packed 3D tissue construct. In our study, we have demonstrated uniform staining in 3D tumor spheroids in both static and perfusion conditions (Figure 3b and Figure 4a). This enabled us to estimate cell viability using a single cross-sectional image of the spheroid (Figure 3f,g and Figure 4b), which reduced image acquisition time substantially and increased the assay throughput to cope with multiple samples during drug dose-response studies. There are many commercially available cell-impermeant nucleic acid-dyes, such as TOTO, TO-PRO, and SYTO family of cyanine dyes, spanning across a wide range of excitation/emission spectra. This offers users flexibility in choosing the dyes. Most nucleic acid dyes have a low intrinsic fluorescence and only emit strong fluorescent signals when bound to nuclei acid, thereby ensuring a high signal-to-noise ratio during image acquisition. More importantly, by specifically labeling the cell nuclei instead of the cytoplasm, one can easily segment and count individual cell nuclei during image processing even in 3D spheroids or organoids, where cells are densely packed together and in direct contact with each other. These features underpinning the QuantICV assay enable a highly customizable method to accurately quantify cell viability in miniaturized 3D tissue constructs. However, it is important to note that the QuantICV assay relies on access to confocal microscopy or other microscopy platforms that enable 3D optical sectioning. Additional factors such as transparency of the microfluidic substrates as well as the thickness of the tissue sample should also be taken into consideration for good image resolution.

The accuracy and sensitivity of the QuantICV assay were validated by comparing the drug responses of two patient-derived tumor cell lines in various culture configurations against a conventional bulk metabolic assay (i.e., MTS assay). IC_50_ values calculated from the dose-response curves provided a quantitative metric for comparison between the two assay methods as well as between the cell lines and culture configurations. The IC_50_ values obtained using the QuantICV assay tallied with those estimated using the MTS assay in both 2D and 3D cultures (Figure 2b and Figure 3c,d). This indicated that our proposed assay can potentially be used in standard cell culture applications as well. The QuantICV assay can not only reliably measure differences in the drug responses between the metastatic and parental OSCC tumor cells, but also variations when the tumor cell lines were subjected to different culture conditions. Using the QuantICV assay, we could measure an increase in sensitivity to Gefitinib in both parental and metastatic OSCC lines when they were maintained in 3D versus 2D cultures. This agreed with a previous study, which reported that OSCCs in 3D cultures expressed higher levels of EGFR and therefore showed increased sensitivity to EGFR inhibitors [38,39]. We further evaluated the feasibility of the QuantICV assay to perform cell viability quantification on microfluidic 3D cultures. Our observation that microfluidic cultures showed higher drug sensitivities compared to static cultures concur with previous literature reports [30,31] and further elucidated the potential attractiveness of microfluidic platforms for more physiological-relevant models. For more complex microfluidic multi-organ systems, where multiple cell culture chambers are fluidically connected [13,33], the QuantICV assay can independently determine the cell viability of individual culture chambers without modification to the microfluidic setup. In contrast, bulk cell viability assays, such as LDH and MTS, rely on analyzing the perfusate, which is circulated between the different cell compartments, and therefore cannot determine the cell viability of individual cell types [7,33]. This makes the assay compatible with a wide range of microfluidic cell culture systems.

## 5. Conclusions

In conclusion, the quantitative image-based cell viability (QuantICV) assay provides an accurate and reliable method for quantifying cell viability in microfluidic 3D tissue cultures, which are not amenable to conventional bulk viability assays. This assay will facilitate future translation of microfluidic tissue models for drug testing applications.

## Supplementary Materials

The following are available online at https://www.mdpi.com/2072-666X/11/7/669/s1. Figure S1: Microfluidic device for the 3D perfusion tissue culture of patient-derived (HN137) OSCCs; Figure S2: Process flow for the silicon mold fabrication process foe the microfluidic cell culture devices; Figure S3: Microfluidic device fabrication flow chart for 3D perfusion tissue culture; Figure S4: Confocal images of Orthogonal projected image of microfluidic 3D culture devices treated with 1.33 stained µM of gefitinib without collagenase treatment before staining process. Scale bar = 100 µm.
