# Supplementary material for: Quantitative Image-Based Cell Viability (QuantICV) Assay for Microfluidic 3D Tissue Culture Applications
Source: Micromachines (Basel). 2020 Jul 9;11(7):669. doi: 10.3390/mi11070669 (PMC7407956; doi:10.3390/mi11070669)
Supplement: Supplementary file 1 [file micromachines-11-00669-s001.pdf]

Supplementary Materials: Quantitative Image-Based Cell Viability (QuantICV) Assay for Microfluidic 3D Tissue Culture Applications

Louis Jun Ye Ong , Liang Zhu, Gabriel Jenn Sern Tan and Yi-Chin Toh

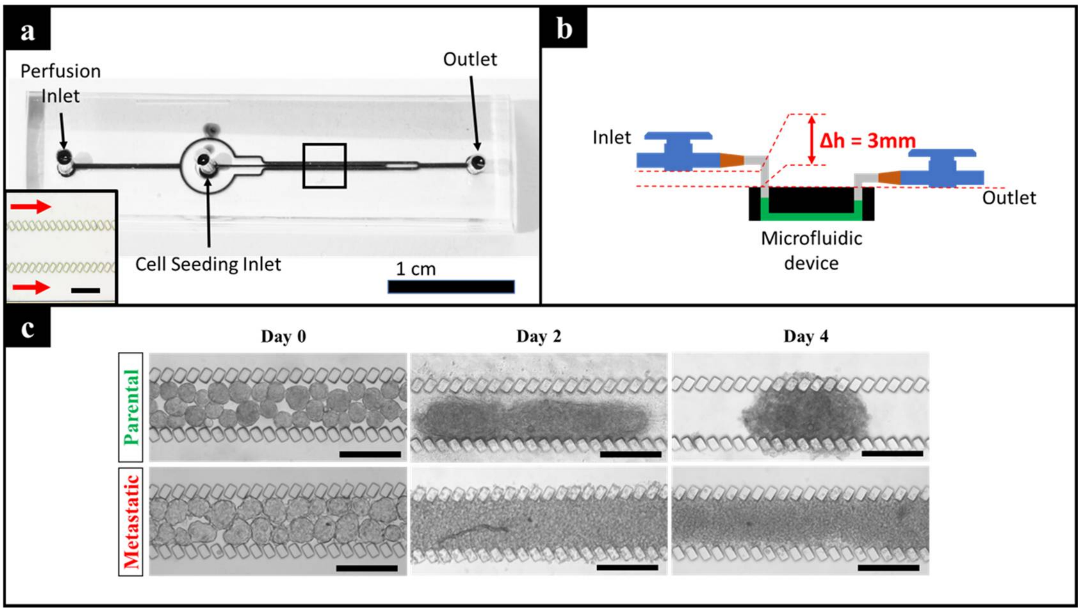

Figure S1. Microfluidic device for 3D perfusion tissue culture of patient (HN137) -derived OSCC. (**a**) PDMS microfluidic culture device with a common outlet and separate inlets for cell seeding and medium perfusion. Scalebar = 1 cm. Insert: Microscopic view of the microfluidic devices with central cell culture compartment line with micropillar array and two perfusion channels. Red arrows indicate the direction of medium flow. Scalebar = 200 μm. (**b**) Pump-free setup of the microfluidic culture for the 3D culture of OSCC. (**c**) Transmission images of parental and metastatic HN137 OSCC in microfluidic devices. Scalebar = 200 μm.

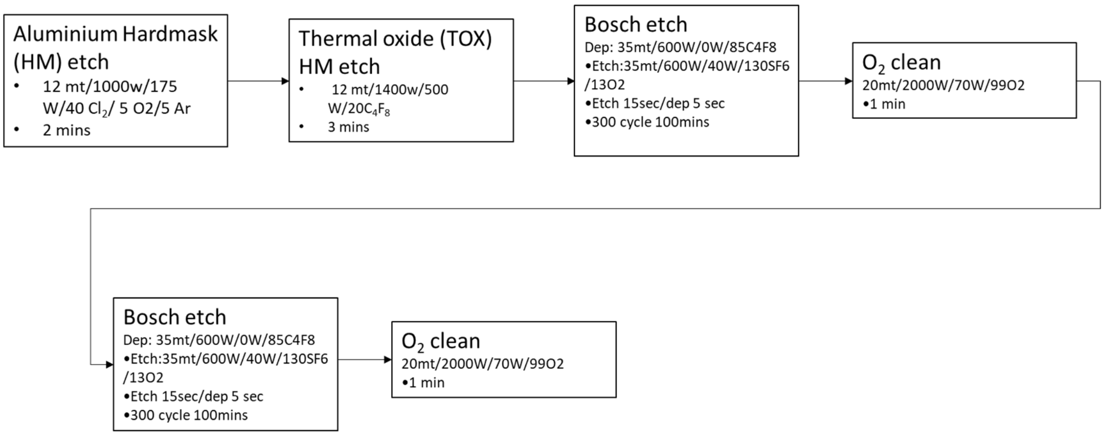

Figure S2. Process flow for the silicon mold fabrication process foe the microfluidic cell culture devices.

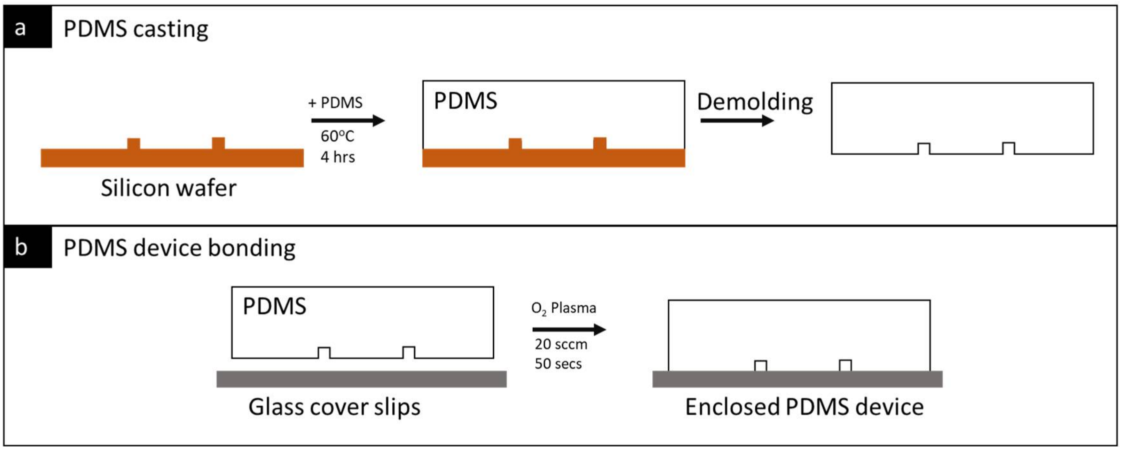

Figure S3. Microfluidic device fabrication flow chart for 3D perfusion tissue culture**.** (**a**) A PDMS device is first fabricated by casting PDMS mixture at 10:1 elastomer: curing agent ratio before curing at 60 °C for 4 h. After curing, the PDMS device is demolded. (**b**) the demolded PDMS is then plasma treated along with glass cover slips with oxygen plasma at 50 sccm for 50 s before permanent bonding is done by pressing the PDMS devices and glass cover slips together to form enclosed channels.

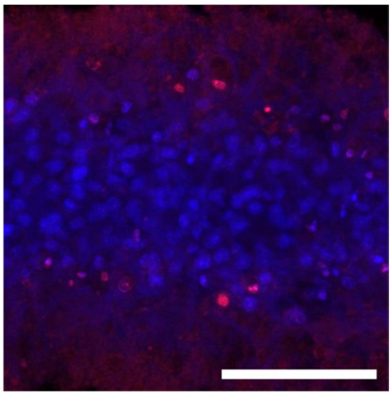

Figure S4. Confocal images of Orthogonal projected image of microfluidic 3D culture devices treated with 1.33 stained µM of gefitinib without collagenase treatment before staining process. Scale bar = 100 µm.
